# Variability in Maize Seed Bacterization and Survival Correlating with Root Colonization by *Pseudomonas* Isolates with Plant-Probiotic Traits

**DOI:** 10.3390/plants13152130

**Published:** 2024-08-01

**Authors:** Melani G. Lorch, Claudio Valverde, Betina C. Agaras

**Affiliations:** 1 Laboratory of Physiology and Genetics of Plant Probiotic Bacteria (LFGBBP), Centre of Biochemistry and Microbiology of Soils, National University of Quilmes, Bernal B1876BXD, Argentina; mglorch@gmail.com (M.G.L.); valverdecl@hotmail.com (C.V.); 2 National Scientific and Technical Research Council (CONICET), Buenos Aires C1425FQB, Argentina

**Keywords:** bioinput, maize seed coating, inoculant, pre-inoculation, bacterial recovery

## Abstract

Seed treatment with plant growth-promoting bacteria represents the primary strategy to incorporate them into agricultural ecosystems, particularly for crops under extensive management, such as maize. In this study, we evaluated the seed bacterization levels, root colonization patterns, and root competitiveness of a collection of autochthonous *Pseudomonas* isolates that have demonstrated several plant-probiotic abilities in vitro. Our findings indicate that the seed bacterization level, both with and without the addition of various protectants, is specific to each *Pseudomonas* strain, including their response to seed pre-hydration. Bacterization kinetics revealed that while certain isolates persisted on seed surfaces for up to 4 days post-inoculation (dpi), others experienced a rapid decline in viability after 1 or 2 dpi. The observed differences in seed bacterization levels were consistent with the root colonization densities observed through confocal microscopy analysis, and with root competitiveness quantified via selective plate counts. Notably, isolates *P. protegens* RBAN4 and *P. chlororaphis* subsp. *aurantiaca* SMMP3 demonstrated effective competition with the natural microflora for colonizing the maize rhizosphere and both promoted shoot and root biomass production in maize assessed at the V3 grown stage. Conversely, *P. donghuensis* SVBP6 was detected at very low levels in the maize rhizosphere, but still exhibited a positive effect on plant parameters, suggesting a growth-stimulatory effect during the early stages of plant development. In conclusion, there is a considerable strain-specific variability in the maize seed bacterization and survival capacities of *Pseudomonas* isolates with plant-probiotic traits, with a correlation in their root competitiveness under natural conditions. This variability must be understood to optimize their adoption as inputs for the agricultural system. Our experimental approach emphasizes the critical importance of tailoring seed bacterization treatments for each inoculant candidate, including the selection and incorporation of protective substances. It should not be assumed that all bacterial cells exhibit a similar performance.

## 1. Introduction

Rhizobiomes or rhizomicrobiomes [1,2] are primarily constituted of a subset of the bulk soil microbiota, and significantly influenced by the seed microbiome [3]. In recent years, there has been a growing interest in the seed microbiomes of staple food crops, such as maize, wheat, and rice, aimed at enhancing food safety and seed quality, as well as exploiting the natural microbial diversity carried by seeds and their plant-growth promoting potential [4,5]. Most research has focused on endophytic seed microbiomes, with limited consideration of the epiphytic seed microbiome [6,7,8]. In all instances, crop genotype emerges as the main factor affecting microbial composition of those microbiomes, while environmental factors play a comparatively minor role [4,7,9]. Generally, a core microbiome can be described, which is rich in specific bacterial genera, such as *Pantoea*, *Enterobacter*, *Sphingomonas*, *Pseudomonas*, *Acinetobacter*, and *Rhodococcus* sp. [3,9,10]. Regarding seed epiphytes, a recent study has indicated that maize seeds exhibit a lower natural microbial load on their surfaces than barley and wheat, with the Proteobacteria group being the most abundant [10]. This finding opens the possibility of bioaugmenting maize seeds at the planting stage through the introduction of suitable plant-growth promoting microorganisms (PGPMs) to facilitate early colonization of the seedling root.

Biological seed treatments have historically been the predominant method for ensuring high densities of introduced microbial inoculants in crop rhizospheres, mimicking the application process of agrochemical products [11,12,13]. This approach has been shown to improve crop production across various geographical conditions, climates, and crop types, among other factors [14,15]. It is particularly applicable to extensive crops that are directly sown into the soil, such as maize, which ranks among the top cultivated crops worldwide, with Argentina being one of the six leading maize-producing countries [16]. The concept behind seed bacterization is to position relatively high amounts of the PGPM cells in the spermosphere, thereby facilitating the establishment of the microbes within the rhizosphere and enhancing root colonization and the intimate interaction between plants and bacteria, which is critical for promoting plant growth [12,17,18]. Furthermore, there is a rising demand from seed companies and growers for access to pre-inoculated seeds that can be stored for weeks or months before sowing [11]. The technology associated with these methods has primarily been developed for legume rhizobial inoculants rather than for other PGPMs [19,20,21]. For rhizobial inoculants, it has been demonstrated that the initial concentration of bacterial cells in the inoculum is directly associated with the number of legume nodules formed and the resulting crop yield [22,23,24].

Challenges related to the survival of microorganisms on seed surfaces, compatibility with agrochemical applications, and the limited tolerance of PGPMs to prolonged exposure to elevated temperatures during the storage of pre-inoculated seeds prompt ongoing exploration for novel additives and application alternatives, such as desiccation protectants, biopriming techniques, film-type coatings, or seed pelleting [12,25]. Significant advancements have been achieved in the development of adjunct substances for formulations based on rhizobia, including sucrose, trehalose, polyethylene glycol, carboxymethyl cellulose, gum arabic, glycerol, xanthan, and other compounds that essentially protect bacteria from desiccation and improve adherence to seeds; they have been tested with varying levels of efficacy [26,27,28]. Trehalose, along with other non-reducing disaccharides, acts as a protectant and protein stabilizer when accumulated in bacterial cells, forming a glass-like sugar matrix that effectively replaces the water hydrogen bonds [29,30,31]. Therefore, to promote its accumulation under various stress conditions, microorganisms activate trehalose synthesis pathways, and/or suppress trehalose catabolism [29,32,33,34]. The combination of this disaccharide with polyvinylpyrrolidone (PVP) enhances the viscosity of the suspensions, potentially improving cell adherence to seeds. PVP retains significant amounts of water, maintains moisture around the cells, and slows the bacterial drying rate [35]. Additionally, the combined effects of certain protective additives, such as trehalose–PVP mixtures, have been reported for specific bacterial species, including genetically modified *Pseudomonas* [35,36,37].

Soybean seeds that are pre-inoculated with commercial rhizobial suspensions can be stored for up to 15 days prior to planting in a cool, well-ventilated location. It has been observed that a seed treatment performed 7 days before sowing improves yields compared to untreated controls [38]. For *Pseudomonas*—non-sporulating Gram-negative bacteria like rhizobia, that are sensitive to abrupt environmental changes such as desiccation—some of these additives have also been evaluated. Seed inoculations with suspensions supplemented with methylcellulose, carboxymethyl cellulose, trehalose, or glycerol have proven effective in wheat, cotton, tomato, and sugar beet [39,40,41,42]. Conversely, comparative studies analyzing the survival of *Pseudomonas* strains in seeds of extensive crops, with or without the addition of these substances, have not yet been conducted, unlike studies with *B. japonicum* [26]. Only in sterilized onion seeds conserved at 4 °C has it been demonstrated that *P. fluorescens* F113 survives for 70 days post-inoculation [43]. Given that, for practical reasons, liquid inoculants are generally favored by producers for seed application, and maintaining high levels of bacterial viability during pre-inoculation until sowing is ideal [13,43]; it would be pertinent to assess the capacity of candidate *Pseudomonas* isolates to remain viable in pre-bacterized seeds if new bioinputs are to be developed.

Therefore, beyond merely describing the plant-probiotic potential that can be assessed in vitro, studying the bacteria–plant interaction throughout all stages (inoculation/competition/colonization/execution of probiotic properties) is essential for optimizing the PGPM performance in agricultural settings, thereby identifying which step is the primary bottleneck for each candidate. However, there are limited studies regarding the performance of seed bacterization and/or the implication of the bacterization levels on root colonization and competitiveness for non-rhizobial microorganisms like *Pseudomonas*. For *Bacillus*, a positive correlation was observed between wheat seed bacterization density and root competitiveness in studies utilizing disinfected seeds inoculated with lyophilized bacteria and carboxymethyl cellulose as an additive [44]. Conversely, for *Stenotrophomonas maltophilia* and *Pseudomonas fluorescens* isolates, no correlation was found between bacterization levels of disinfected wheat seeds and root competitiveness in field assays using also carboxymethyl cellulose as an additive [45]. In addition to the contrasting evidence for the same crop with different bacterial isolates, there are no reports in the literature of such studies using non-disinfected seeds.

Members of the *Pseudomonas* genus are recognized as keystone components of rhizospheric microbiomes, contributing to plant growth and health [46,47,48]. In our laboratory, we have obtained and characterized a set of *Pseudomonas* isolates exhibiting plant-probiotic potential [49]. This collection encompasses representatives of various species, including *P. protegens, P. chlororaphis, P. soli*, and *P. donghuensis*, some of which have been recently documented in the literature, particularly concerning their biological control capabilities against phytopathogenic fungi [49,50,51]. Additionally, we have observed that certain isolates could improve the grain yield in wheat and maize through field assays [52]. Therefore, to optimize the development of bioformulations based on these microorganisms, the aims of this study are: (1) to analyze the bacterization level achieved through seed inoculation using non-disinfected seeds and doses recommended for agricultural producers; (2) to monitor the survival capacity of each tested strain on pre-bacterized seeds in the presence of protectant additives; (3) to characterize their root colonization pattern with confocal microscopy; (4) to evaluate their root competitiveness, quantifying their density in maize seedlings grown in soil in the presence of competing microflora.

## 2. Results

### 2.1. Chromosomal Tagging of Pseudomonas Isolates for Facilitating Enumeration in Bacterized Seeds and Visualization in Colonized Roots under Axenic Conditions

To analyze the levels of bacterization in non-disinfected seeds by plate count on selective media, and to visualize the bacterial cells on root surfaces to assess root colonization patterns of our *Pseudomonas* isolates under non-axenic conditions, we introduced antibiotic resistance markers and genes encoding fluorescent proteins. The Tn*7* mutagenesis approach proved optimal for these modifications without altering the genetic background of the *Pseudomonas* strains [53,54,55]. Selected Tn*7* derivatives maintained physiological characteristics, PGPR traits, and growth rates indistinguishable from wild type isolates (Figures S1 and S2). Growth curves with trehalose demonstrated that only SVBP6 and SVMP4 were unable to utilize this non-reducing disaccharide as a carbon source (Figure S2). Therefore, trehalose could exclusively act as an osmoprotectant for these isolates, maintaining membrane and protein integrity [56]. We also confirmed the stable integration of Tn*7* cassettes in the absence of an antibiotic selection pressure (Table S1), ensuring that selective counts from bacterized seeds accurately reflected the load of viable cells after different varying incubation times.

### 2.2. Seed Bacterization Levels Were Strain-Dependent

**Figure 1:**
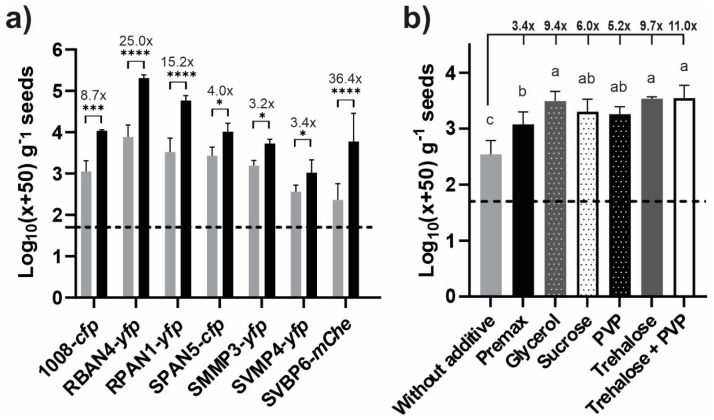
Variability in maize seed bacterization levels and protective effects within our *Pseudomonas* collection at the time of inoculation (0 dpi). The data represent transformed CFU values using the formula log_10_(x + 50), where x is the corresponding CFU value. This transformation is pertinent for statistical comparisons of data sets including CFU null data [57]. Dashed lines indicate the log_10_ value corresponding to a transformed null CFU count. (**a**) Recovery of culturable *Pseudomonas* from maize seeds inoculated in the presence (black) or absence (grey) of the commercial bacterial protectant Premax^®^ (Rizobacter Argentina S.A., Pergamino, Argentina). Asterisks indicate statistically significant differences between treatments with or without Premax^®^ (ANOVA with LSD-Fisher multiple comparison test; * *p* < 0.05, *** *p* < 0.001, **** *p* < 0.0001). Numbers above treatment bars indicate fold increase in bacterial recovery for treatments with Premax^®^ compared to the corresponding control. (**b**) Effect of different additives on recovery of SVMP4-*yfp* from maize bacterized seeds. Different letters indicate statistically significant differences (ANOVA with LSD Fisher’s multiple comparison test, *p* < 0.05). Numbers above bars indicate the fold increase in bacterial recovery for treatments with additives compared to the corresponding control.

The evaluation of seed bacterization levels at the time of inoculation (0 days post-inoculation, dpi) demonstrated that incorporating the Premax^®^ additive in the bacterial suspensions significantly enhanced the recovery of culturable cells from the seeds. Premax^®^ improved the bacterial recovery for all isolates compared to control treatments, albeit to varying extents (Figure 1a). The improvement ranged from 3.2× to 39.4×, indicating genuine protective and/or adhesive effects of the additive on bacterial cells. Specifically, RBAN4-*yfp* and RPAN1-*yfp* showed higher bacterization recovery values than the reference strain 1008-*cfp*, both with and without the additive (Figure S3). Conversely, SVMP4-*yfp* achieved lower bacterization levels than the reference 1008-*cfp*, both with and without the additive (Figure S3). Consequently, we tested various additive compounds with SVMP4-*yfp* to enhance its maize seed bacterization. All the tested additives improved SVMP4-*yfp* recovery, with trehalose, glycerol, and a trehalose–PVP mixture showing the highest values, increasing bacterization by 2.8×, 2.7× and 3.2×, respectively, compared to the Premax^®^ levels (Figure 1b). Germination remained unaffected by treatments or storage conditions.

### 2.3. Pre-Hydration of Maize Seeds Improved Recovery of Culturable Bacterial Cells

**Figure 2:**
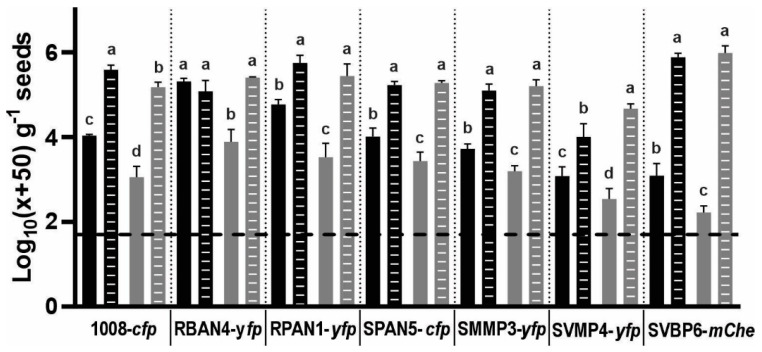
Pre-hydration of maize seeds before bacterization increases the recovery of culturable *Pseudomonas* cells at the time of inoculation (0 dpi). Treatments compared include those with (black) and without (grey) the addition of the commercial protectant Premax^®^, and with (dashed bars) or without (full bars) pre-hydration treatment (see main text for details). See Figure 1 legend for the reference about data transformation. Dashed line indicates the log_10_ value corresponding to a transformed null CFU count. Different letters indicate statistically significant differences between treatments for each isolate (ANOVA with LSD Fisher’s multiple comparison test, *p* < 0.05).

Recommended seed treatment doses involve minimal liquid quantities (a few microliters per seed), incorporating both chemical and biological inputs to prevent initiation of the germination process. Consequently, bacterial cells contacting seed surfaces may undergo desiccation shock due to the rapid liquid uptake by the seeds, potentially reducing cell culturability and/or viability. Given the favorable effect of additives on seed bacterization levels, we investigated whether our isolates’ performance could be impacted by seed dryness. Pre-hydrated seeds before inoculation nullified the protective benefits of the Premax^®^ for all isolates, resulting in higher bacterization levels for almost all strains (except for RBAN4) compared to non-soaked seeds inoculated with the addition of Premax^®^ (Figure 2). Particularly, pre-hydration significantly enhanced the recovery of culturable SVMP4-*yfp* cells compared to the Premax^®^ treatment alone (Figure 2). The inoculant dose corresponds to 5 µL of the bacterial suspensions per gram of seeds, containing 10^4^–10^6^ CFU. In our assays, we recovered between 0.3 and 9.1% of the bacteria incorporated in the presence of Premax^®^, whereas these percentages did not exceed 0.51% when no additive was used (Table S2). These recovery rates substantially improved with seed pre-hydration, reaching 7.0 to 12.0% with Premax^®^ (Table S2). However, RBAN4-*yfp* showed consistent recovery values irrespective of pre-hydration or the Premax^®^ addition (Figure 2), with no increase in recovery percentages. Even isolates losing the additive’s effect with pre-hydration reached slightly higher recovery percentages without the additive when the seeds were pre-hydrated (Table S2). Particularly, recovery of SVMP4-*yfp* cells from pre-hydrated seeds without Premax^®^ reached 29.47% of the inoculated bacteria, contrasting with the 1.8% recovery obtained in the presence of Premax^®^ (Table S2).

### 2.4. Bacterization Levels of Maize Seeds Strongly Declined up to 4 dpi

**Figure 3:**
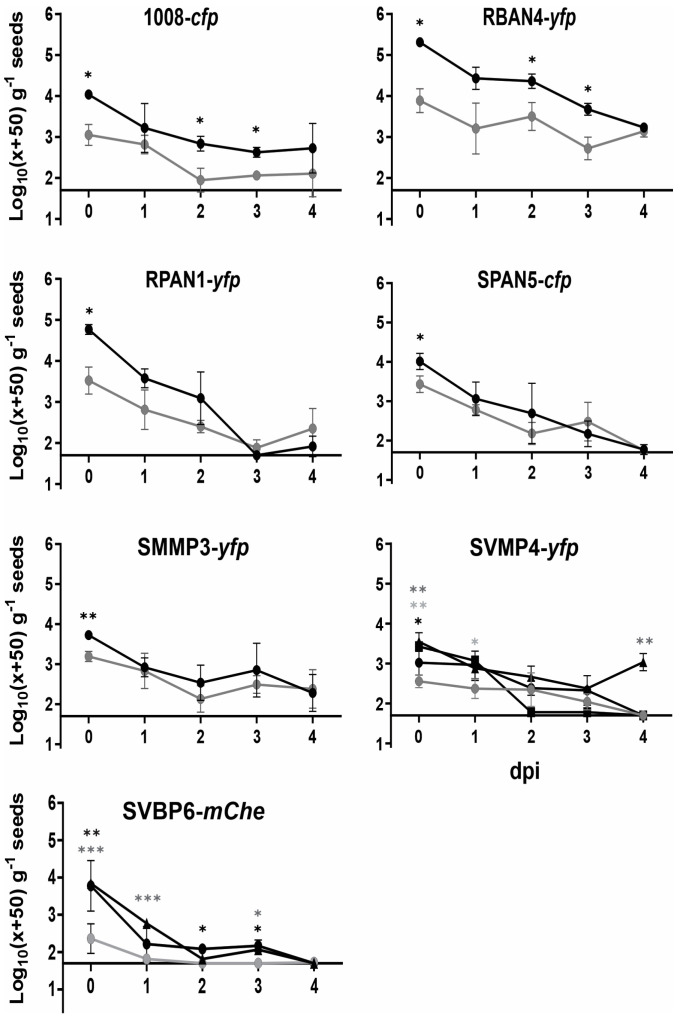
Time course recovery of *Pseudomonas* isolates from bacterized maize seeds is strain-dependent, showing a decline in the additive effect over time for some isolates. Bacterization levels (CFU) were determined in seeds sampled daily for up to 4 days after inoculation with (black) or without (grey) additives. Premax^®^ treatment is denoted with black circles; trehalose–PVP mixture treatment is shown with triangles; and glycerol with squares. See Figure 1 legend for the reference about data transformation. Asterisks indicate statistically significant differences between additive and control treatments on the same day (Two-way ANOVA with LSD Fisher’s multiple comparison test; *** *p* < 0.001; ** *p* < 0.01; * *p* < 0.05). Black asterisks denote statistically significant differences for Premax^®^ treatment; dark grey asterisks denote differences for trehalose–PVP treatment; light grey asterisks denote differences for glycerol treatment.

Once we identified the optimal seed bacterization treatment for each isolate, we studied the decay kinetics of recovered culturable cells for a period of up to 4 dpi (Figure 3). Given the practical implications for agricultural producers, who often prefer seed treatments shortly before sowing, we chose a time frame comparable to a standard business week to evaluate bacterization kinetics. RBAN4-*yfp* showed the best performance, with the highest recovery values and a significant positive effect by the Premax^®^ additive throughout the entire experiment (Figure 3). The reference strain 1008-*cfp* performed similarly, although the bacterial cell recoveries were lower (Figure 3). For the remaining isolates, the protectant effect of Premax^®^ was observed only at 0 dpi; subsequent daily CFU counts exhibited a decaying trend that was statistically indistinguishable regardless of the use of the Premax^®^ additive (Figure 3). This pattern was evident even for RPAN1-*yfp*, which initially exhibited higher bacterization levels at 0 dpi but decayed more rapidly than 1008-*cfp* thereafter (Figure 3). For SVMP4-*yfp* and SVBP6-*mChe*, the presence of the trehalose–PVP mixture improved the bacterial recovery, but only SVMP4-*yfp* retained recoverable cells up to 4 dpi. The bacterial cell suspensions stored under conditions identical to the bacterized seeds showed no significant viability decline up to 4 dpi (Table S3).

### 2.5. Colonization Patterns on Maize Seedlings Were Particular and Congruent with Bacterization Levels

**Figure 4:**
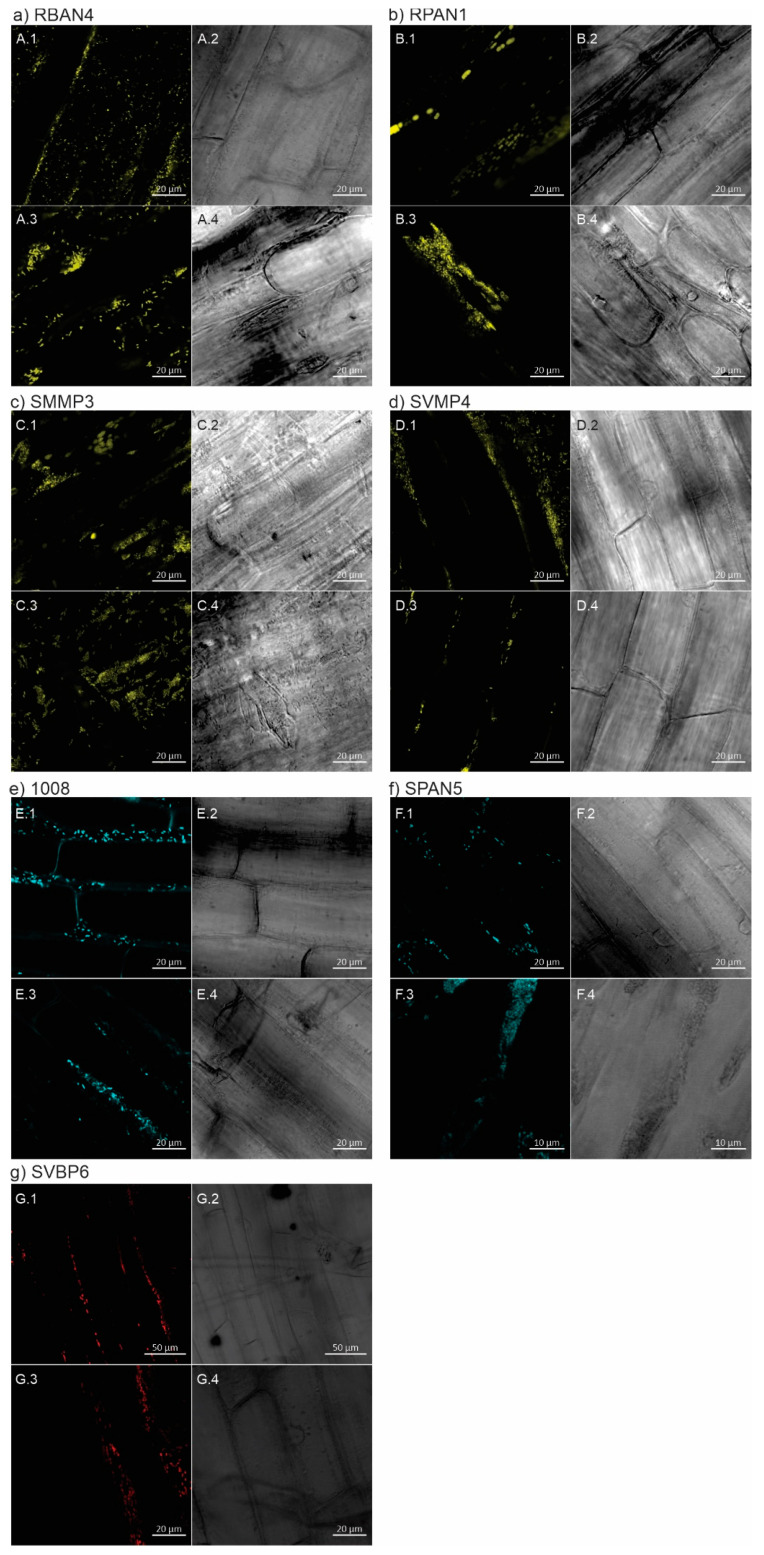
*Pseudomonas* isolates can achieve different colonization patterns on the root surface of maize seedlings after seed bacterization. Early root colonization (9 days after inoculation) was analyzed by confocal fluorescence microscopy of *Pseudomonas* isolate derivatives expressing different fluorescent proteins: eYFP (**a**–**d**), eCFP (**e**,**f**) and mCherry (**g**). All images were captured at the elongation zone of the maize roots (1 cm above the root tip) using a 20× objective and 4× digital zoom, except for (**g**) (**1**,**2**) (2× zoom) and (**f**) (**3**,**4**) (40× objective and 4× zoom). In all cases, pictures from right panels (**2**,**4**) correspond to transmitted light microscopy and those from left panels (**1**,**3**) to confocal fluorescence microscopy. Images are representative of colonization patterns on all the observed plant roots (2–4 replicates per treatment). Control treatments were also observed, obtaining in all cases images without any fluorescent bacterial cells. Scales are indicated with a white bar on each image, corresponding to 20 µm, except for (**f**) (**3**,**4**) (10 µm) and (**g**) (**1**,**2**) (50 µm).

The observation of the maize seedlings under confocal microscopy allowed us to analyze the different colonization patterns on the root surfaces (Figure 4). We demonstrated that our isolates achieved acceptable levels of root colonization of the maize seedlings, preferentially occupying the plant cell junctions. However, we could discern three main configurations of bacterial cells on the epidermal surfaces: string-like arrangements (Figure 4e–g); microcolonies dispersed on the surface (Figure 4b,c); or a combination of both, microcolonies and string-like arrangements (Figure 4a,d,f). RBAN4-*yfp*, which demonstrated the best seed bacterization performance (Figure 1), also displayed the highest root colonization level, with dense bacterial populations on root surfaces (Figure 4a), similar to colonization densities of the reference strain 1008 (Figure 4e). In contrast, SVMP4-*yfp* showed inconsistent patterns and low colonization levels across all replicates (Figure 4d). This observation aligns with the poor performance of this isolate during maize seed bacterization at 0 dpi (Figure 1).

### 2.6. Maize Root Competitiveness Is Consistent with Seed Bacterization Levels, although Growth Promotion Effects Were Also Detected in Less Competitive Isolates

**Figure 5:**
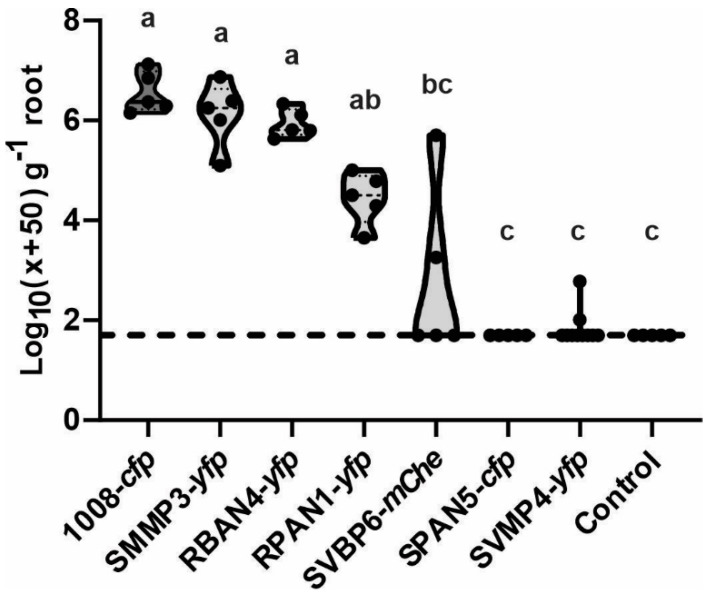
*Pseudomonas* isolates showed different root competitiveness during the early colonization of maize roots in the presence of natural soil microflora. Root colonization was quantified by selective plate counts on S1 media supplemented with the corresponding antibiotic for every *Pseudomonas* derivative. See Figure 1 legend for reference about data transformation. Dashed line indicates the log_10_ value corresponding to a transformed null CFU count. Data was corrected to express the values by the dry root weight. Different letters indicate statistically significant differences between treatments (Kruskal–Wallis non-parametric test, with the uncorrected Dunn’s multiple comparison test).

**Figure 6:**
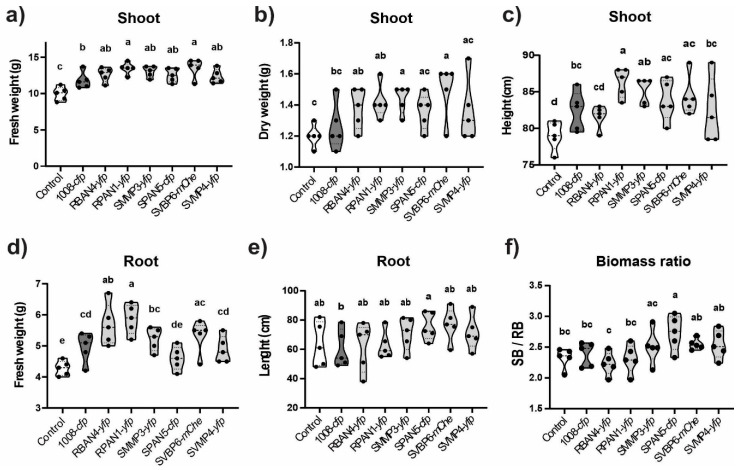
Bacterial inoculation improved several plant parameters of maize up to the V3 growth stage. After one month of incubation, we measured the shoot biomass, fresh (**a**) and dry (**b**); the shoot height (**c**); the fresh root biomass (**d**); the root length (**e**); and the shoot/root biomass ratio (SB/RB, **f**). For all the measured data, we included a non-inoculated control (white) and the reference treatment 1008 (dark grey). Different letters indicate statistically significant differences between treatments (ANOVA with LSD Fisher’s multiple comparison test, *p* < 0.05).

After one month of plant growth in natural soil, we assessed the root competitiveness of the isolates by quantifying their abundance in a maize rhizosphere. The load of culturable, heterotrophic, mesophilic bacteria showed values between 5.7 × 10^8^ and 3.5 × 10^7^ CFU per gram of dry root (Table S4). We found significantly higher root colonization abundance for those strains that had shown the highest bacterization levels on the maize seeds at 0 dpi (Figure 5). Notably, SPAN5-*cfp* was the only isolate undetected in the maize rhizosphere (Figure 5) despite showing good seed bacterization levels (Figure 1). SVBP6-*mChe* and SVMP4-*yfp*, which had low seed bacterization rates (Figure 1 and Figure 3), were also heterogeneously detected in rhizospheric samples (Figure 5). However, when we evaluated the plant-growth promoting effect of seed inoculation with these *Pseudomonas*, we observed improvements across most measured parameters (Figure 6), even for those isolates poorly detected in the rhizosphere, such as SPAN5-*cfp* and SVBP6-*mChe* (detection limit: <500 CFU g^−1^ root). Particularly, the RBAN4-*yfp*, RPAN1-*yfp*, SMMP3-*yfp*, and SVBP6-*mChe* treatments significantly enhanced the shoot biomass (Figure 6a,b), even though the RBAN4-*yfp* treatment did not affect plant height (Figure 6c). Moreover, we found a positive correlation between the shoot biomass (fresh or dry), plant height, and root biomass (Figure S4). Analysis of root growth revealed increased root fresh weight by the *Pseudomonas* inoculation (Figure 6d), while root length remained similar across all the treatments (Figure 6e). Finally, the ratio of shoot to root biomass (SB/RB) indicated that SPAN5-*cfp* was the only treatment that significantly promoted shoot development without increasing root biomass, although SVBP6-*mChe* and SVMP4-*yfp* also showed similar performances (Figure 6f).

## 3. Discussion

### 3.1. Variability in Maize Seed Bacterization among PGPMs from the Same Bacterial Genus

It is generally assumed that different types of bacterial cells can adhere to and survive on seed surfaces with similar success [12]. Consequently, this aspect is not typically studied for inoculant candidates. In this work, we demonstrated distinct behaviors among isolates from various species within the same genus when applied to the surfaces of the same hybrid of maize seeds (Figure 1a). Comparing these isolates to a commercial *Pseudomonas* strain marketed as a bioinput for extensive crops [58], we found that some isolates achieved better, comparable, or worse bacterization levels, with CFU densities ranging from 3 to 5.5 log_10_ per gram of maize seeds, depending on the isolate (Figure 1). Given an average surface area of 0.75 µm^2^ per rod-shaped bacterial cell from the *Pseudomonas* genus [15], and an average maize seed surface area of 1250 mm^2^ for the variety used in our experiments, the bacterial densities achieved imply a maximum coverage of 15–45% of the maize seed surface. Similar findings were noted across different crop seeds, suggesting an irregular spatial distribution of cells on seed surfaces [59,60]. While even higher levels of maize seed bacterization (over 10^6^ CFU per g) have been reported for a *Pseudomonas* isolate like the strain *P. putida* mt-2, our results generally align with other studies testing various *Pseudomonas* isolates using different inoculation techniques, highlighting limitations in achieving high bacterial densities on maize seed surfaces [61,62]. In previous studies, the adherence of *Pseudomonas* to seeds has been linked to the synthesis of cell surface proteins, like LapA and LapF, as well as flagella-mediated processes [63,64,65,66,67]. Consequently, differential biochemical and/or expression properties of these surface macromolecules in the tested isolates may impact their ability to adhere to seeds. Consistent with our seed bacterization results, the evaluation of adhesion capabilities to abiotic surfaces (e.g., polystyrene) has shown that RBAN4, RPAN1, SPAN5, and SMMP3 adhered more efficiently to such surfaces compared to SVBP6 and SVMP4 [49].

### 3.2. Enhancement of Seed Bacterization with a Commercial Bacterial Protectant Additive Was Also Strain-Dependent

Seed bacterization success can be significantly enhanced by incorporating a cell protectant and/or adhesive compounds into inoculant formulations [68,69,70]. Our study demonstrates that different protectants indeed improve bacterization performance of the tested isolates (Figure 1). The efficacy of Premax^®^ in enhancing the bacterial recovery from the seeds was evident at 0 dpi (Figure 1a), and up to 3 dpi (Figure 3) for RBAN4-*yfp*, a member of the *P. protegens* subgroup (Table 1). However, at 4 dpi this enhancement was no longer observed, with approximately 10^3^ CFU per gram of seeds recovered regardless of the presence of the additive. This behavior, unique to this isolate among those tested, suggests a physiological feature enabling it to maintain viability and culturability throughout our evaluation period. Such persistence could facilitate pre-inoculation strategies for bioinputs formulated with RBAN4. Given that members of the *P. protegens* species are characterized by their multiple plant-growth promotion abilities, mainly associated with biocontrol [71], preserving viable cells at sowing becomes crucial for further colonizing the rhizosphere. Notably, the *P. protegens* strain CHA0 has been reported to enter a viable but non-culturable (VBNC) state under stressful conditions and in particular soil microbial habitats [72,73]. Therefore, RBAN4 might similarly enter this state under the potentially stressful conditions offered by seed surfaces. Future investigations employing VBNC detection techniques [74] could elucidate if RBAN4 cells persist longer with higher viable cell densities on maize seed surfaces.

### 3.3. The Incorporation of Trehalose and PVP Ameliorates Seed Bacterization for Isolates Experiencing Higher Dehydration Stress Upon Seed Inoculation

For all tested strains, pre-hydration (imbibition) of the seeds enhanced the bacterization levels at 0 dpi more effectively than the addition of protectants (Figure 2). This suggests that the *Pseudomonas* isolates studied herein undergo dehydration stress on the seed surfaces, leading to decreased viability in the absence of protectants. This effect was particularly pronounced for SVMP4-*yfp*, which exhibited the lowest bacterization levels (Figure 1 and Figure 2). Previous studies have shown that the environmental model microorganism *P. putida* KT2440, a plasmid-cured derivative of the strain mt-2, is highly sensitive to desiccation stress, inducing a VBNC state under such conditions [75,76]. However, this sensitivity can be mitigated by introducing the *otsBA* operon from *E. coli*, which is involved in trehalose synthesis, enabling *P. putida* KT2440 to accumulate this osmolyte in its cytoplasm [37,77]. From our pseudomonads collection, strains SVBP6 and SVMP4 lack the ability to metabolize trehalose (Figure S2), and these isolates showed the lowest bacterization recovery and a pronounced decline in their survival on the seeds up to 4 dpi (Figure 1 and Figure 3). Furthermore, supplementing NYB media with 20 mM trehalose did not alter their bacterization performances. Supporting these experimental results, genomic analyses of SVBP6 revealed the absence of homologous genes associated with trehalose metabolism (*treP*, *treA* and *treR*). Although we have not sequenced the genome of SVMP4, these trehalose-related genes are not present in the genomes of other *P. soli* isolates. However, with a similar approach, we found homologous genes related to trehalose synthesis, *treS* and *treY*/*Z.* Therefore, we suggest that both isolates are not capable of internalizing trehalose molecules, and that under the stressful experimental conditions of seed bacterization, these isolates might not activate trehalose synthesis or that the amount of trehalose synthetized is not sufficient to support their viability, as it occurs in *P. putida* KT2440 [37]. Nevertheless, we observed a better seed bacterization performance of SVMP4-*yfp* with the addition of a trehalose–PVP mixture compared to Premax^®^ (Figure 1b), whereas SVBP6-*mChe* performed similarly with both additive treatments (Figure 3). This suggests that the efficacy of this mixture for these two isolates relates to modifying external conditions that favor their adherence and reduce dehydration shock. Previous reports have demonstrated the beneficial effects of externally added trehalose in non-trehalose-producing cells under stress [37,56,78,79,80]. Additionally, the protective role of the trehalose–PVP mixtures has been reported for various bacteria [35,36,37]. Given that both SVBP6 and SVMP4 belong to the extensive *P. putida* subgroup [81], our results suggest that these isolates might also enter the VBNC state after the inoculation process in response to the desiccation shock on the seed surfaces, potentially explaining the decline in culturable counts. Further investigation using VBNC approaches would contribute to explore this possibility [76,82].

### 3.4. The Beneficial Effects of Plant-Growth Promoting Microorganisms Might Not Solely Stem from Root Colonization and Competitiveness

It is conventionally assumed that root colonization is essential for the effective promotion of plant growth by microorganisms, as direct contact with plant tissues enables PGPMs to exert their beneficial effects on plants while utilizing root exudates as nutrient sources [18]. Cell motility and biofilm formation are associated with a superior colonization performance [83,84,85], and we have previously demonstrated that all our isolates display these abilities in vitro [49]. As microorganisms can exhibit distinct spatial arrangements along the root, occupying specific niches [67,86,87], we used confocal microscopy to analyze root colonization patterns of our *Pseudomonas* isolates (Figure 4). The presence of bacterial cells on the rhizoplane confirmed their ability to exit seed coats and colonize the emerging roots. We observed a differential localization of bacterial cells on maize root epidermal surfaces (Figure 4). Typically, microorganisms show a preference for cell junctions of the root epidermis, a region proposed as highly permeable for root exudates through the apoplastic pathway [88,89]. Notably, the commercial reference strain 1008-*cfp* and our derivative SVBP6-*mChe* primarily occupied cell junctions, leaving outer surfaces of epidermal cells largely unoccupied (Figure 4e,g). In contrast, SMMP3-*yfp* and RPAN1-*yfp* predominantly occupied outer epidermal surfaces (Figure 4b,c). Other isolate derivatives (RBAN4-*yfp*, SVMP4-*yfp*, and SPAN5-*cfp*) displayed a mixed arrangement (Figure 4a,d,f). These differential colonization patterns likely influence their ability to effectively exert biocontrol activity, as competition for niches and nutrients is one of the strategies involved in plant protection by *Pseudomonas* [87,90]. From our collection of autochthonous *Pseudomonas* isolates with plant-probiotic traits [49,52], RBAN4-*yfp,* RPAN1*-yfp*, and SMMP3-*yfp* exhibited the highest colonization densities on maize radicles (Figure 4). These isolates have shown superior bacterization levels, with RBAN4-*yfp* and RPAN1*-yfp* significantly outperforming the control treatment 1008-*cfp* (Figure S3). Thus, root colonization densities appear correlated with seed bacterization levels, as previously shown for other *Pseudomonas* isolates on alfalfa [91].

The confocal microscopy findings agree with our root competitiveness assay, where these three isolates were the most abundant in rhizospheric samples after one month of maize growth, i.e., up to the V3 stage, despite competition from soil microflora (Figure 5). However, the plant-growth promotion observed post-seed inoculation extended to all the *Pseudomonas* isolates, regardless of their root competitiveness (Figure 6). In previous results under axenic conditions, we also observed that SMMP3 significantly improved the early growth of maize [49]. Moreover, this isolate increased maize grain yield in experimental field assays when inoculated alone or in combination with a *Trichoderma* fungal isolate [52]. Therefore, SMMP3′s robust root surface colonization likely contributes to its positive effects on plant growth and crop yield. Conversely, we also noted an enhanced shoot length, shoot biomass, and root biomass in plants treated with SVBP6-*mChe* (Figure 6b–d), despite its heterogeneous and low detection in rhizospheric samples (Figure 5) and microscopy-based colonization (Figure 4g). Overall, confocal microscopy revealed reasonable bacterial colonization densities in the maize root elongation zone for all tested strains, indicating an efficient root colonization performance by these isolates through the strategy of seed bacterization before sowing. However, intriguingly, after one month of competition with the natural microflora, certain isolates such as SVMP4, SVBP6, and SPAN5, were unable to maintain detectable colonization levels in the maize rhizosphere (Figure 5). The improvement in seed bacterization achieved by SVMP4 with the addition of the trehalose–PVP mixture (Figure 1b) seemed insufficient to enable this isolate to compete with the natural soil microflora (Figure 5). Although we did not detect these isolates in the rhizospheric samples of maize plants at the V3 stage, our results suggest that the interaction between bacteria and plant organs during early plant development stages stimulates plant growth, even after the decline in the inoculated strain density on the root. In this regard, a recent study has shown that the initial contact between a plant-growth promoting *Bacillus subtilis* strain and melon seeds modifies the seedling development process, leading to a positive effect on adult plants [92]. Furthermore, it demonstrated that some *Bacillus* derivative strains, despite not colonizing the melon radicle, maintain their promoting effect on adult plants due to the production of specific metabolites such as the amyloid protein TasA and the lipopeptide fengycin, which alter plant metabolism [92]. Therefore, we propose that some of our *Pseudomonas* isolates may similarly impact maize growth through the production of specific metabolites. However, further experiments are necessary to challenge this hypothesis thoroughly.

## 4. Materials and Methods

### 4.1. Bacterial Strains, Growth Condition and Plant Species

Six bacterial isolates from our *Pseudomonas* collection [49] and their respective Tn7 tagged strains were utilized in this study (Table 1). *P. pergaminensis* 1008, the active principle of the commercial inoculant Rizofos^®^ (Rizobacter Argentina s.a., RASA, Pergamino, Argentina), recommended for maize inoculation in Argentina [58], served as a reference. Bacterial strains were stored in 20% *w*/*v* glycerol at −80 °C, and routinely cultured on nutrient agar (NA, tryptone soy agar 40 g L^−1^; yeast extract 5 g L^−1^, Biokar, Cedex, France) or nutrient yeast broth (NYB, nutrient broth 20 g L^−1^; yeast extract 5 g L^−1^, Biokar Cedex, France). Incubation was carried out at 28 °C (except for *Escherichia coli* strains, which were grown at 37 °C), with agitation at 200 rpm for liquid cultures. The selective medium Gould’s S1 [93] was used to specifically quantify the endogenous CFU load of *Pseudomonas* isolates of non-disinfected, untreated seeds. Media were supplemented with antibiotics as needed (Gm, gentamycin 20 µg mL^−1^; Km, kanamycin 25 µg mL^−1^; Ap, ampicillin 100 µg mL^−1^; Sm, streptomycin 100 µg mL^−1^; all from Sigma Aldrich, Darmstadt, Alemania). The commercial target crop was *Zea mays* (maize) and seeds belonged to the variety KM 8701 VIP3 or KM 87 VIP3 (KWS Argentina s.a., Balcarce, Argentina). Due to their larger size compared to typical maize seeds, 2–3 seeds per gram were used.

### 4.2. Chromosomal Tagging of Pseudomonas Isolates

Isolates were tagged with fluorescent proteins and an antibiotic resistance gene using a Tn7 transposition-based system, which inserts a single copy of the antibiotic resistance gene along with a gene encoding a fluorescent protein (eYfp, yellow; eCfp, cyan; mCherry, red) at a neutral chromosomal site [53,94]. Briefly, equal volumes from overnight cultures of *E. coli* SM10λpir/pUX-BF13, *E. coli* HB101/pME497, the corresponding *E. coli* carrying the eyfp, or ecfp delivery vector (MT102) or the mCherry delivery vector (DH5α), and each target *Pseudomonas* isolate (Table 1) were combined in a tetraparental mating mix on the edge of a NA plate and incubated for 4 h at 37 °C. This conjugation mixture was resuspended in 1 ml of NYB and 100 µL were spread onto M9 minimal medium agar plates [95] supplemented with citrate 0.2% as a carbon source and the corresponding antibiotic from the Tn7 cassette [53,96]. After incubation at 28 °C for 48 h, 3 or 4 colonies of each *Pseudomonas* isolate were streaked onto new Gould’s S1 agar plates containing the appropriate antibiotic. Colony PCR was conducted to confirm the correct Tn*7* insertion using specific oligonucleotides Tn*7*R109 and Tn*7*-*glmS* [53]. To verify that the tagged strains retained the same phenotype as the wild type, the PGPM properties demonstrated in vitro by the wild type isolates [49] were re-evaluated, and growth curves on M9-glucose (20 mM) and M9-trehalose (10 mM) were performed and compared between the variants and wild type strains. The growth curve with trehalose aimed to assess if this disaccharide could be used as an osmoprotectant if not metabolized by either strain [41]. Stability of the genomic insertion was also analyzed by subculturing the variants for 5 days without the selective pressure of the antibiotic, and then plating onto agar plates with or without the antibiotic.

**Table 1:** Bacterial isolates and plasmids used in this work.

***Pseudomonas* Isolates (Wild Type)**			
**Name**	**Taxonomical Affiliation ^a^**	**Origin**	**Reference**
RBAN4	*P. protegens* ^b^	Pasture rhizosphere, Natural Environment, Bengolea, Córdoba, Argentina	[49]
SMMP3	*P. chlororaphis* subsp. *aurantiaca*	Bulk soil from soybean plot, Monte Buey, Córdoba, Argentina	[49]
SPAN5	*P. chlororaphis*	Bulk soil from pasture plot, Pergamino, Buenos Aires, Argentina	[49,97]
SVMP4	*P. soli*	Bulk soil from pasture plot, Viale, Entre Ríos, Argentina	[49,98]
SVBP6	*P. donghuensis*	Bulk soil from soybean plot, Viale, Entre Ríos, Argentina	[49,50]
RPAN1	*P. chlororaphis* subsp. *piscium*	Pasture rhizosphere, Natural Environment, Pergamino, Buenos Aires, Argentina	[49]
1008	*P. pergaminensis*	Wheat rhizosphere from a productive plot, Pergamino, Buenos Aires, Argentina	[58]
***Pseudomonas* derivative strains**			
**Name**		**Relevant genetic and/or phenotypic features**	**Reference**
RBAN4-*yfp*		RBAN4::*att*Tn*7*-*eyfp*; Km^r^ Sm^r^	This study
SMMP3-*yfp*		SMMP3::*att*Tn*7*-*eyfp*; Km^r^ Sm^r^	This study
SPAN5-*cfp*		SPAN5::*att*Tn*7*-*ecfp*; Km^r^ Sm^r^	This study
SVMP4-*yfp*		SVMP4::*att*Tn*7*-*eyfp*; Km^r^ Sm^r^	This study
SVBP6-*mChe*		SVBP6::*att*Tn*7-mCherry*; Gm^r^	This study
RPAN1-*yfp*		RPAN1::*att*Tn*7-eyfp*; Gm^r^	This study
1008-*cfp*		1008::*att*Tn*7*-*ecfp*; Km^r^ Sm^r^	This study
***Escherichia coli* strains**			
**Strains**		**Relevant genetic and/or phenotypic features**	**Reference**
SM10		*thi-1, thr, leu, tonA, lacY, supE, recA*::RP4-2-Tc::Mu, λ*pir*. Km^r^	[99]
HB101		Laboratory strain. K12 derivative. F^−^ Pro^−^ Gal^−^ Rec^−^ Sm^r^	[100]
DH5α		Laboratory strain. K12 derivative. F^−^ *recA1*^−^ *endA1*^−^ *lacZ*ΔM15	[101]
MT102		Laboratory strain. *ara*D*139* D*(ara-leu)7697 Δlac thi hsdR* derivate of *E. coli* K-12 substrain MC1000. Sm^r^ Rif^r^ Azide^r^	[102]
**Plasmids**			
pUX-BF13		Helper plasmid for Tn*7*-based transposon mutagenesis containing the transposition functions; R6K-replicon; Ap^r^	[103]
pME497		Mobilizing plasmid; IncP-1, Tra; RepA(Ts); Ap^r^	[104]
pME9407		Delivery plasmid for mini-Tn*7-mCherry*; pME3280a carrying *mCherry* placed under Plac control; Ap^r^ Gm^r^	[94]
miniTn*7*(Km, Sm)P_A1/04/03_–*ecfp*-a		Delivery plasmid for mini-Tn*7-ecfp*; pUC19 derivative carrying *ecfp* under a constitutive promoter (Plac derivative). Not replicative in *Pseudomonas.* Ap^r^ Km^r^ Sm^r^	[53]
miniTn*7*(Km, Sm)P_A1/04/03_–*eyfp*-a		Delivery plasmid for mini-Tn*7-eyfp*; pUC19 derivative carrying *eyfp* under a constitutive promoter (Plac derivative). Not replicative in *Pseudomonas.* Ap^r^ Km^r^ Sm^r^	[53]

^a^ For SVBP6, RBAN4, SMMP3, and 1008, the taxonomy affiliation was performed by phylogenomic analyses. For SVMP4, SPAN5, and RPAN1, the affiliation was performed with taxonomical markers (16S rDNA, oprF and rpoB genes, see [49]; ^b^ The taxonomy affiliation was recently corrected with an ANI performed with the sequenced genome.

### 4.3. Seed Inoculation and Recuperation of Bacteria from Seeds

To prepare the inoculation mix, *Pseudomonas* tagged strains were grown in Erlenmeyer flasks with rotary agitation with a 5:1 flask/culture volume ratio, for 16 h at 28 °C. Bacterial cells were centrifuged at 5000 rpm and 4 °C for 10 min. The pellet was washed twice with saline solution (NaCl 0.85% *w*/*v*) and normalized to an OD_600_ of 1.0 for seed inoculation. Viable cell counts were determined by drop plate count onto NA supplemented with the corresponding antibiotic (Table S1). Non-disinfected maize seeds were inoculated following the recommended dose of Rizofos^®^ inoculant for maize at the start of the experiment (7 mL kg^−1^). Initially, we assessed bacterization in the presence or absence of the commercial additive Premax^®^ (RASA, Pergamino, Argentina). For this purpose, the inoculation mix was prepared with 2 mL kg^−1^ of the additive and 5 mL kg^−1^ of the bacterial suspension. Control treatments replaced additive volume with saline solution. When Premax^®^ did not enhance seed bacterization levels, we evaluated alternative additives: (i) trehalose 1M [37]; (ii) polyvinylpyrrolidone (PVP) 1.5% *w*/*v* [40]; (iii) a mixture of both, trehalose and PVP; (iv) glycerol 20% *w*/*v* [40]; and (v) sucrose 20% *w*/*v* [105]. The ratio between bacterial suspension and additive was consistent across all cases (5 mL kg^−1^ for the bacterial suspension and 2 mL kg^−1^ for the additive). The total volume of each inoculation mix was added to seeds in a sterile plastic container and vigorously shaken by hand for 1.5 min.

Immediately after inoculation, bacteria were recovered from seeds and quantified on selective solid medium (day 0). For this, 5 g of seeds were suspended in 15 mL of saline solution in 50 mL screw-cap tubes. After vortexing for 10 s and immersing the tubes in a sonication bath (40 kHz, 160 W, Testlab TB04, Bernal, Argentina) for 10 min to promote detachment of bacteria from seed surface and dispersion of bacterial aggregates. Tubes were centrifuged at 50× *g* at room temperature for 1 min to remove coarse material. The supernatant containing bacteria was transferred to sterile 50 mL tubes, and serial dilutions were plated in triplicate onto Gould’s S1 plates with the corresponding antibiotic from the Tn*7* cassette for quantification of culturable pseudomonads recovered from seeds using the drop count method [106].

Bacterization decay kinetics were assessed for up to 4 days post inoculation (dpi), with daily recovery and enumeration of bacteria. During this period, seeds were incubated in a plant growth chamber (Ingelab Argentina, I-501 PF, Llavallol, Argentina) in darkness with temperature cycling from 24 °C to 13 °C (16/8 h, respectively). As a control for the intrinsic cell survival, daily viable cell counts were conducted in the bacterization suspensions with OD_600_ = 1.0, maintained under the same storage condition as the bacterized seeds. Additionally, the effect of seed bacterization on germination was assessed by sowing seeds on 1% (*w*/*v*) water agar plates and determining the proportion of germinated seeds out of the total sown.

### 4.4. Bacterization of Pre-Hydrated Seeds

To determine if seed water content influenced bacterization levels, seeds were first soaked in sterilized, deionized water for 16 h at 120 rpm, dried with absorbent paper to prevent dripping; and then inoculated following the same procedure described in Section 4.3, both with and without the addition of Premax^®^. Bacteria were recovered from seeds immediately after inoculation (0 dpi) and quantified by drop plate count on selective medium, as previously described.

### 4.5. Visualization of Bacterial Root-Colonization Patterns by Confocal Laser Scanning Microscopy (CLSM)

Maize seeds were surface disinfected with 70% *v/v* ethanol for 1 min, followed by treatment with 1.1% *w*/*v* NaClO for 10 min [107]. Then, seeds were washed 5 times with sterile distilled water for 2 min each and finally dried on a paper towel for 15 min in a laminar flow. Seed inoculation was carried out as described above, using the optimal additive for each strain. Control treatment involved a mixture of saline solution and the respective additive at the specific usage proportion. After inoculation, seeds were placed in sterile cotton-stoppered glass tubes containing 30 mL of semi-solid (0.5% *w*/*v* agar) Jensen’s mineral solution [108] with 50 mM ok KNO_3_ and incubated for 9 days in a plant growth chamber in darkness, under the conditions mentioned above. Roots segments from seedlings were cut into small pieces (around 1 cm) from 1 cm of root tips and visualized by scanning different focal planes of the surface using a laser scanning confocal microscope (Zeiss LSM 880 with Airyscan, Leloir Institute, Buenos Aires, Argentina) with either a Plan-Apochromat 20×/0.8 or C-Apochromat 40×/1.2 objectives, and the electronic zoom to improve visualization. Excitation wavelengths were between 405 and 458 nm for isolates tagged with eCFP; 488 nm for those tagged with eYFP; or 543 nm for mCherry. Emission wavelengths were between 463 nm and 498 nm for eCFP; between 515 nm and 574 nm for eYFP; or between 583 nm and 675 nm for mCherry (Table 1).

### 4.6. Root Competitiveness and Plant-Growth Promotion of Maize Grown from Bacterized Seeds

To analyze if the *Pseudomonas* isolates can occupy the rhizosphere of maize from bacterized seeds competing with the natural soil microflora, we designed a greenhouse assay. We used 1 L pots filled with sterilized perlite and natural soil mixed in 1:1 volume ratio, with one plant per pot and five plants per treatment (n = 5). The soil was obtained from a pristine environment located in Llavallol, Buenos Aires Province, Argentine (34°47′12.8″ S, 58°26′28.6″ W), and sieved by a 2 mm mesh prior to use. Non-disinfected maize seeds (variety KM 87 VIP3) were inoculated with the optimal additive for each isolate (Premax^®^ for RBAN4-*yfp*, SMMP3-*yfp*, RPAN1-*yfp*, SPAN5-*cfp*, and the reference 1008-*cfp*; trehalose–PVP mixture for SVMP4-*yfp* and SVBP6-*mChe*) or treated with saline solution for the negative control. Pots were incubated under greenhouse conditions with temperature and humidity ranging from 20 to 24 °C and from 60 to 80%, respectively; and a day–night cycle of 16–8 h (supplemented with artificial light to mitigate seasonal variations in the natural photoperiod). Substrate humidity was maintained at field capacity with sterilized distilled water applied 3 times a week. After 1 month of incubation (V3-V4 maize stage), plants were harvested and the root systems were gently shaken to remove large, adhered soil particles and obtain the “rhizospheric sample” (i.e., the complete root system with the small soil particles tightly adhered to its surface). Rhizospheric samples were collected and kept at 4 °C until processing as previously described [57] to quantify bacterial colonization using the drop plate assay [106]. Total heterotrophic, mesophilic, and culturable bacteria were quantified by plating onto diluted tryptone soy agar (TSA 10%, Biokar, Cedex, France) supplemented with cycloheximide 100 µg mL^−1^ (Anedra, Buenos Aires, Argentina) to inhibit fungal growth [57]. Specific counts for each inoculated isolate were performed on S1 agar plates supplemented with cycloheximide 100 µg mL^−1^ and the corresponding antibiotic for each isolate. For isolates resistant to both Km and Sm (Table 1), plates were supplemented with both antibiotics to prevent growth of soil microflora with natural Km resistance. Colony counts were conducted after 48 h of incubation at 28 °C. At the time of plant harvest, we measured the height of the aerial part, the length of the primary root, and the fresh and dry weight of the aerial part, as well as the fresh weight of the whole root system after removing large and loosely adhered soil particles. A subsample of the root system was dried to determine bacterial colonization density based on dry root biomass.

### 4.7. Statistical Analyses

To compare the daily recovery of bacteria from seeds, three replicate samples were used (n = 3). *In planta* assay under greenhouse conditions was performed with 5 biological replicates. CFU counts were performed on Gould’s S1 in triplicates for each replicate. CFU values were transformed using the formula log_10_(x + 50) prior to statistical analyses, to account for null values [57]. Analysis of variance (ANOVA) or the Kruskal–Wallis non-parametric test were performed as appropriate, followed by the “Least Significant Difference” (Fisher’s LSD) or “Uncorrected Dunn’s” multiple comparison tests, respectively, using GraphPad Prism V. 8.00 for Windows (GraphPad Software, La Jolla, CA, USA, www.graphpad.com) to evaluate significant differences between values. Statistical significance was set at *p* < 0.05.

Due to unsynchronized seed germination, the number of replicate samples for visualizing bacterial root-colonization patterns varied among strains. The number of replicate samples ranged from 2 to 4 seeds per treatment.

## 5. Conclusions

In our study, we characterized the distinct performances of a group of autochthonous plant-probiotic *Pseudomonas* strains concerning their capacities to remain viable and culturable for up to 4 days post-inoculation on the surface of inoculated maize seeds. Our observations revealed significant variability in the ability of isolates to adhere to and persist in the culturable state on maize seeds, with a correlation between root colonization density, root competitiveness, and bacterization levels. Additionally, we demonstrated that bacterial protectants added to the inoculant suspensions promoted seed bacterization, with a differential impact depending on the isolate. Notably, the performance of three isolates from our *Pseudomonas* collection was comparable to that of 1008-*cfp*, a Tn*7* derivative from a strain commercialized as a maize bioferstilizer with demonstrated benefits [58]. This suggests that maize seed bacterization with these isolates could be implemented similarly to achieve crop yield improvements in the field. Finally, we observed that although some isolates did not achieve high root colonization densities in adult plants under natural soil conditions, they still exerted a plant growth promotion effect one month after seed bacterization and sowing. These findings underscore the importance of thoroughly examining these factors when implementing a seed inoculation strategy to introduce PGPMs into agricultural ecosystems. Our results highlight the need to carefully consider seed adhesion and persistence characteristics to optimize the effectiveness of PGPMs in promoting plant growth. Ongoing analyses are also underway to extend our investigation to wheat and soybean seeds, aiming to broaden our understanding of the interactions between *Pseudomonas* strains and diverse plant species within agricultural settings.

## Data Availability

The raw data supporting the conclusions of this article will be made available by the authors on request.

## Supplementary Materials

The following supporting information can be downloaded at: https://www.mdpi.com/article/10.3390/plants13152130/s1, Figure S1: Phenotypic check for the neutrality of mini-Tn*7* cassette integration into the chromosome of wild type *Pseudomonas* isolates; Figure S2: Growth curves of wild type isolates (black symbols) and the corresponding Tn*7* tagged variants (grey symbols) in M9 minimal medium supplemented with 20 mM glucose (triangles) or 10 mM trehalose (circles); Figure S3: Impact of the commercial bacterial protectant Premax^®^ on maize bacterization levels of the *Pseudomonas* isolates at the day of inoculation (0 dpi); Figure S4: Pearson correlation analysis between the plant parameters that were evaluated in the greenhouse experiment; Table S1: Transposon stability under the absence of antibiotic selection pressure; Table S2: Soil properties; Table S3: Cell density of the seed bacterizing suspensions and percentage of culturable bacterial cells recovered from bacterized seeds; Table S4: Strain survival (CFU mL^−1^) in seed bacterizing suspensions stored in parallel and under the same conditions as maize bacterized seeds; Table S5: Rhizospheric abundance of culturable, heterotrophic, mesophilic bacteria.
