# Supplementary material for: Variability in Maize Seed Bacterization and Survival Correlating with Root Colonization by Pseudomonas Isolates with Plant-Probiotic Traits
Source: Plants (Basel). 2024 Aug 1;13(15):2130. doi: 10.3390/plants13152130 (PMC11314135; doi:10.3390/plants13152130)
Supplement: Supplementary file 1 [file plants-13-02130-s001.zip › Supp. figures with captions.pdf]

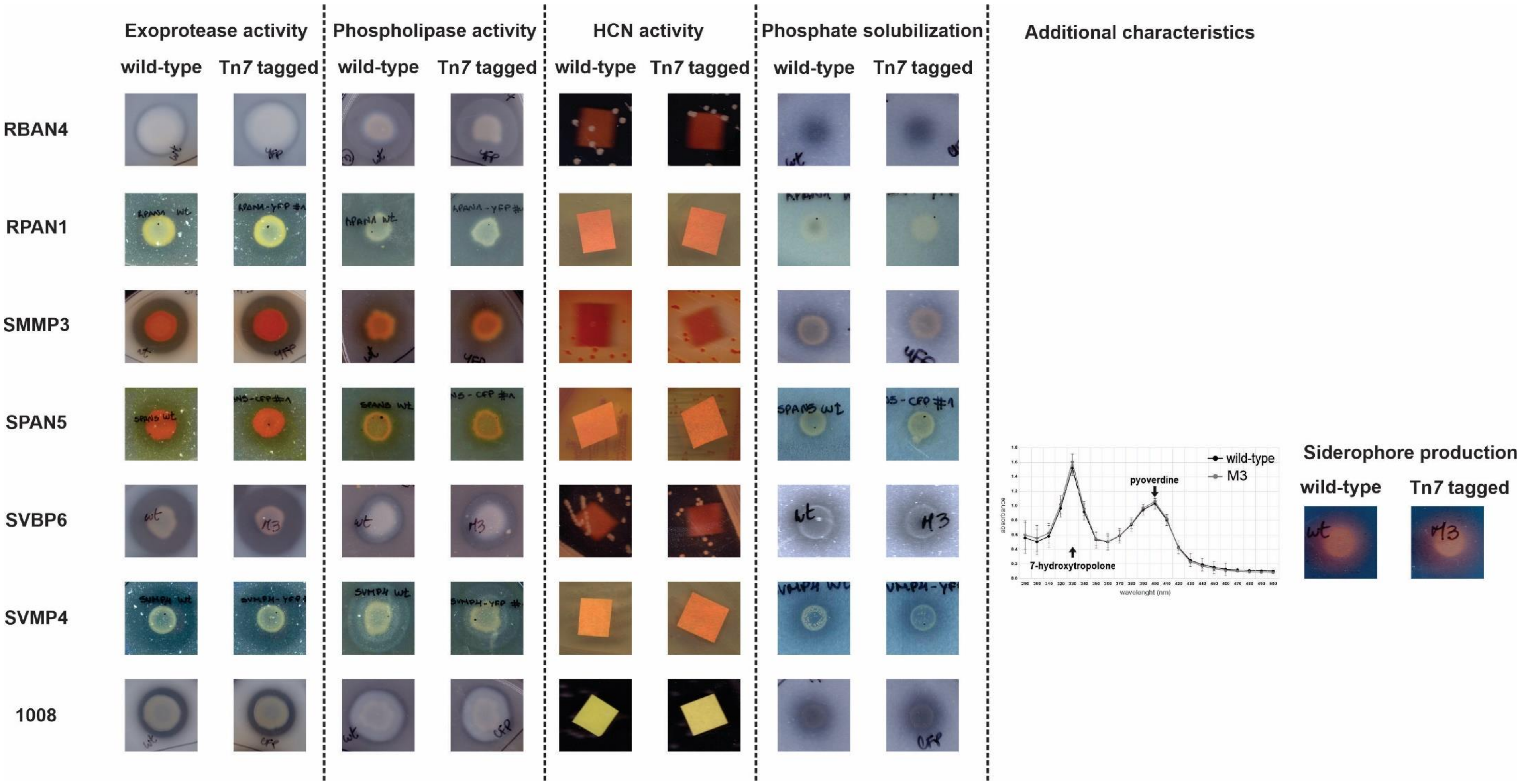

Figure S1. Phenotypic check for the neutrality of mini-Tn*7* cassette integration into the chromosome of wild type *Pseudomonas* isolates. Different plant-growth promoting traits were evaluated *in vitro* to assess whether tagged variants preserved the activities reported previously [46]. Except for the phosphate solubilization assay (plates incubated for 7 days), all the activities were evaluated after 48h of incubation at 28 °C. Milk agar plates were used for the exoprotease activity [105] and egg-yolk agar plates for phospholipase activity [105]. The picric acid assay was used to detect HCN production, evaluating the color change of papers from yellow (negative) to orange (positive, [106]. Phosphate solubilization was evaluated with the NBRIP medium, using $Ca_3(PO_4)_2$ as the phosphate source [107]. For SVBP6, we also evaluated production of 7-hydroxytropolone and pyoverdine by UV-visible spectrometry of King's B culture supernatants [108] and the CAS-agar assay [109]

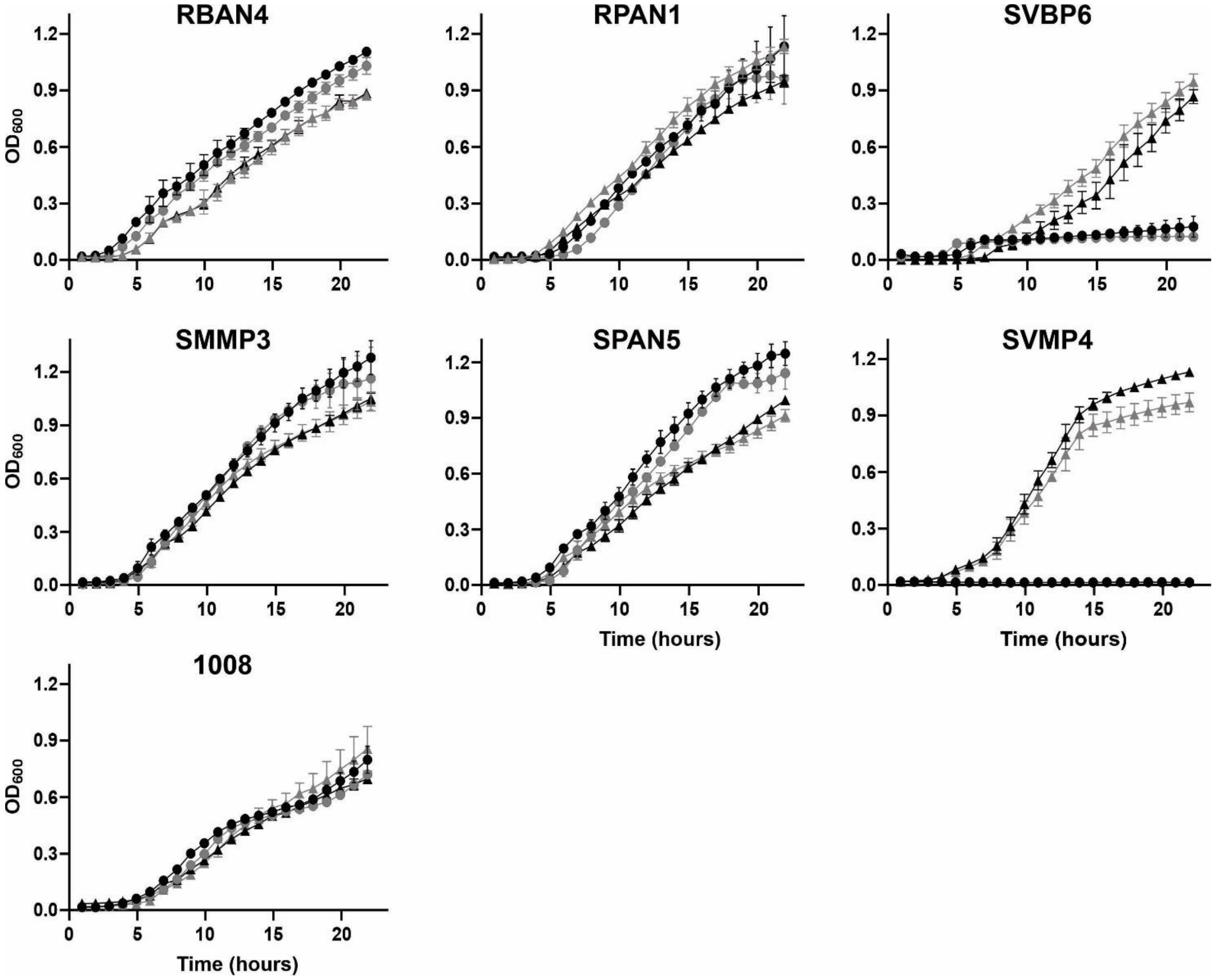

Figure S2. Growth curves of wild-type isolates (black symbols) and the corresponding Tn*7* tagged variants (grey symbols) in M9 minimal medium supplemented with 20 mM glucose (triangles) or 10 mM trehalose (circles). There was no significant difference in the growth performances between the wild-type and the tagged variants. Growth was monitored in a microplate reader (200 µl per well, 180 rpm of agitation, and 28 °C) and the data shown correspond to the average and standard deviation for n = 3 replicates per strain.

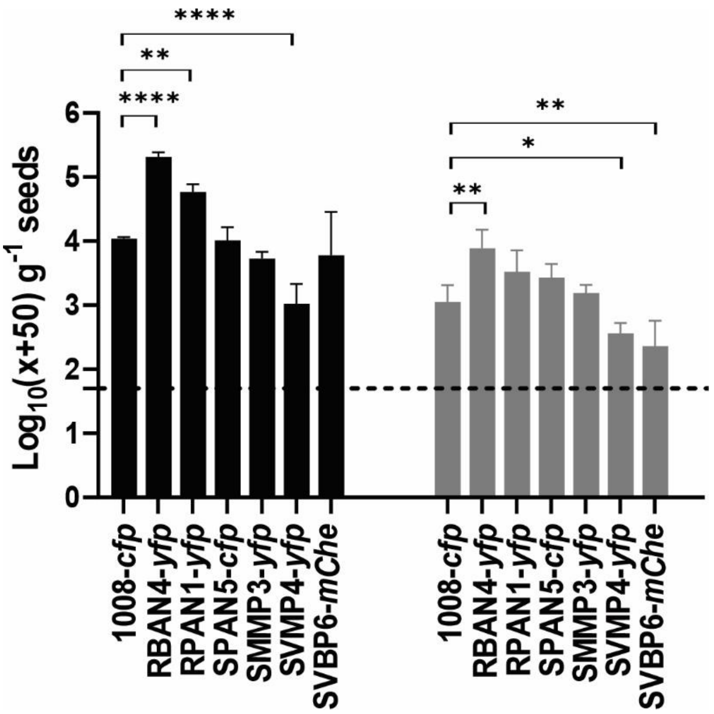

Figure S3. Impact of the commercial bacterial protectant Premax® on maize bacterization levels of the *Pseudomonas* isolates at the day of inoculation (0 dpi). See Figure 1 legend for the reference about data transformation. Dashed lines indicate the log_{10} value corresponding to a transformed null CFU count. Black bars correspond to bacterization levels of isolates inoculated in the presence of Premax®, whereas grey bars correspond to isolates bacterized without Premax® (see main text for details). Asterisks indicate statistically significant differences between isolates of each treatment group (ANOVA with LSD-Fisher multiple comparison test; * means p <0.05, ** means p < 0.01, **** means p < 0.0001).

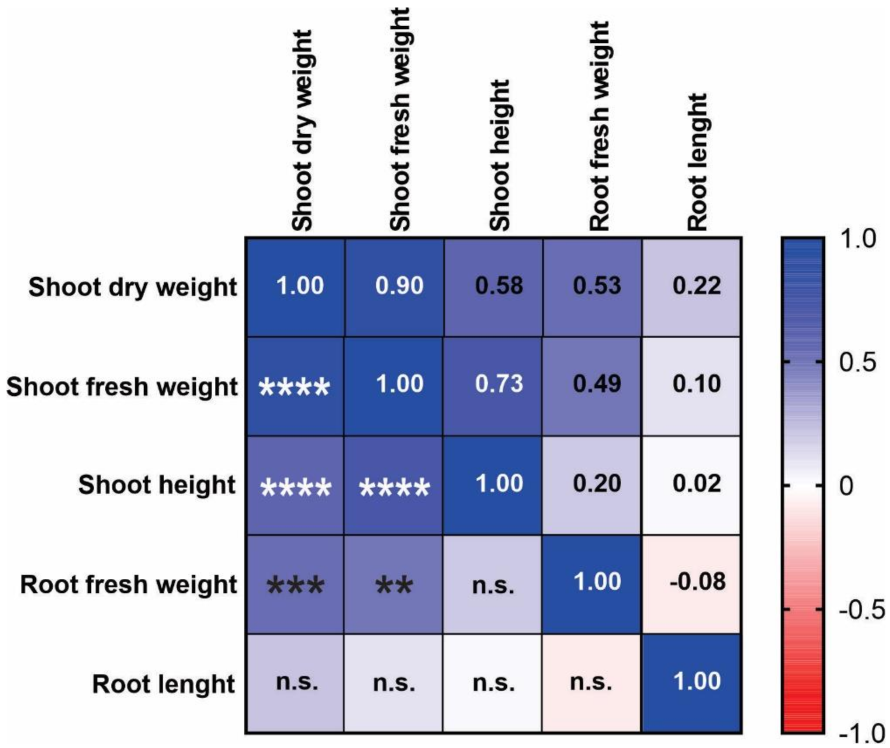

Figure S4. Pearson correlation analysis between the plant parameters that were evaluated in the greenhouse experiment. The upper part of the matrix shows the Pearson coefficient values, whereas the lower part shows the significancy level of the statistics. ** means p < 0.01, *** means p < 0.001, **** means p < 0.0001; n.s., no significant)
